# Differences in BMI obesity measures in a workers compensation population: a cross-sectional study

**DOI:** 10.1097/MS9.0000000000000428

**Published:** 2023-04-01

**Authors:** Mark H. Hyman, Tamra J. Peled, Noah M. Hyman, Jingyi Tan, Xiuqing Guo, Jerome I. Rotter

**Affiliations:** a HymanHealth, Los Angeles, California; b Yeshiva College, New York, New York; c Wharton School of Business, Philadelphia, Pennsylvania; d Department of Pediatrics, The Institute for Translational Genomics and Population Sciences, The Lundquist Institute for Biomedical Innovation at Harbor-UCLA Medical Center, Torrance, California, USA

**Keywords:** BMI, body fat distribution, obesity, race

## Abstract

To assess the accuracy of BMI compared to directly measured dual-energy X-ray absorptiometry percent body fat (DEXA %BF) among a worker compensation population.

**Methods:**

The agreement between BMI and DEXA %BF was assessed by the Pearson correlation coefficient among 1394 evaluable patients over a 5-year period. Sensitivity and specificity were calculated to measure how well BMI can identify true obese and nonobese individuals.

**Results:**

Using at least 30kg/m^2^ to identify obesity, BNI had a specificity of 0.658 and a sensitivity of 0.735. The correlation was better in females at 0.66, compared to males at 0.55, and weaker in older age groups at 0.42, as compared to the youngest age group at 0.59. Overall, 29.8% of the population was reclassified based on their DEXA %BF measures.

**Conclusions:**

In a 5-year cohort worker compensation population, BMI was an inaccurate measure of true obesity.

## Introduction

HIGHLIGHTS

- There is unreliability of the most common measure of obesity in the general population – the BMI.
- A more reliable method is using dual-energy X-ray absorptiometry.
- The presence or absence of obesity also influences injury recovery, morbidity, work productivity, and absenteeism
- Knowing whether someone is truly obese makes a difference in their health and recommendations.

Obesity is an established risk factor for diabetes, cardiovascular disease, obstructive sleep apnea, and early mortality^1^,^2^. The WHO and other societies classically define obesity using BMI. BMI calculations based on height and weight do not consider body mass variability in adults that can occur with different ethnic groups, ages, or sex^3^, except that a different BMI threshold has been recommended for the Asian population^4^.

Although BMI may give some idea of obesity in a general population, it does not take individual variable factors into account. In addition, BMI has not been shown to accurately reflect obesity in special populations, such as active^5^ and retired football players^6^. Multiple statements from the American Heart Association (AHA) emphasize that BMI can overestimate or underestimate obesity as assessed by visceral fat^7^.

Misclassification has significant clinical relevance as patients may not get adequate counseling and other interventions based on false reassurance from a BMI table. Similarly, misclassifying patients as obese can lead to the negative consequence of using unnecessary treatment.

The tendency of BMI to inaccurately identify adiposity in the general population has been seen in other studies^8^,^9^. Quantification of body fat can be done most reliably with a computed tomography scan, MRI, or water displacement^10^; the latter is often viewed as the gold standard^11^. However, these modalities are expensive or generally not easily available. While BMI cutoffs can be inaccurate, dual-energy X-ray absorptiometry (DEXA) has been shown to accurately measure percent body fat against the referenced standards^12^–^14^. Studies have shown that DEXA complements the four-compartment model, which determines body fat based on body density, body volume, DEXA bone mineral values, and total body water^15^. As demonstrated by the AHA, DEXA can now serve as an accessible alternative for body fat calculation in the absence of water displacement. To date, we are not aware of a large cross-sectional assessment of the correlation between BMI and obesity as assessed by DEXA. In this paper, we report our results in an unselected adult worker’s compensation population. Our objective was to determine the correlation in diagnosis of obesity based on the metropolitan life table BMI versus dual-energy X-ray absorptiometry percent body fat (DEXA %BF).

## Methods

The study population consisted of 1394 consecutive patients seen for a worker’s compensation evaluation during the time period of 2016–2021 at a single practice. A medical chart review was conducted for each patient, including their age, sex, weight, height, race, BMI, and direct measurement of percent body fat. This was determined with the DEXA machine (Hologic Discovery model QDR; Hologic Inc., Bedford, Massachusetts, USA); accuracy and validation have been described elsewhere^16^. The categorization of underfat, healthy range, overweight, and obese based on %BF is shown in Table 1. While normal life tables list a BMI of 30 as obese^17^, for the Asian population in our sample, we used a BMI obesity cutoff of 27.5, as has been referenced in other studies^18^. This work has been reported in line with the STROCSS (strengthening the reporting of cohort studies in surgery) criteria^19^, Supplemental Digital Content 1, http://links.lww.com/MS9/A42.

**Table 1:** Directly measured DEXA %BF normal ranges.

Age	Underfat	Healthy range	Overweight	Obese
Women				
20–40	<21	21–33	33–39	>39
41–60	<23	23–35	35–40	>40
61–79	<24	24–36	36–42	>42
Men				
20–40	<8	8–19	19–25	>25
41–60	<11	11–22	22–27	>27
61–79	<13	13–25	25–30	>30

DEXA %BF, dual-energy X-ray absorptiometry percent body fat.

### Data analysis

We calculated the Pearson correlation coefficient *r* to assess the association between quantitative measures of BMI and DEXA %BF. To evaluate the agreement between obesity defined by BMI standards of the WHO and obesity defined by DEXA %BF, we calculated sensitivity and specificity to measure how well BMI can identify true positives and true negatives, respectively, when using DEXA %BF as the ‘Gold Standard.’ ROC curves (receiver operating characteristic curves, Supplementary Figure 1, Supplemental Digital Content 2, http://links.lww.com/MS9/A43) were used to show the performance of BMI at all classification thresholds when predicting obesity defined by DEXA %BF. We also calculated the proportion of overestimate and underestimate of obesity by BMI, sex, and by race. Two-proportions *z* test was used to compare two observed proportions. A two-sided *P* value of less than 0.05 was considered statistically significant. Statistical analyses were performed in R software, version 4.1.0.

## Results

**Figure 1:**
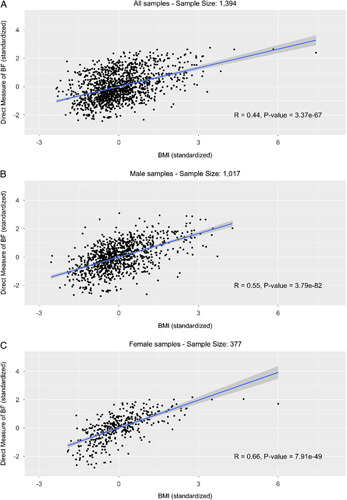
Direct measure of body fat (DEXA %BF) versus BMI by gender (standardized). (A) All samples – sample size: 1394; (B) male samples – sample size: 1017; (C) female samples – sample size: 377.

There was a positive correlation between BMI and DEXA %BF, with a correlation coefficient of 0.44 in 1394 samples in this data set and a corresponding *P* value less than 0.001 (Fig. 1A). There are 12 cases who had BMI values more than 3 SD away from the mean and 2 extremely obese cases who had BMI values more than 5 SD away from the mean. We performed the correlation analysis after removing the 2 outliers and 12 outliers, respectively, and found the correlation estimate is quite robust (0.44 in all samples, 0.43 after removing the 2 extreme outliers, and 0.42 after removing the 12 outliers (Supplementary Table 1, Supplemental Digital Content 3, http://links.lww.com/MS9/A44). Overall, our Worker Compensation cohort showed higher measures of obesity relative to population norms^20^. In all, 802 of 1394(57.5%) people in this data set were obese by BMI and 797 (57.2%) were obese by DEXA %BF. These obesity rates are higher than the national obesity rate which approaches 40%^21^. One reason for this may be the large percentage of public safety personnel in our study, which is one of the most common occupational groups for obesity.

We found that 210 people, 15.1% of the sample size, when directly measured, were not classified as obese, yet according to the BMI calculation, they would have been diagnosed as obese. In addition, BMI underestimated obesity in 205 (14.7%) people that were classified as obese based on their directly measured body fat. From this, we determined that for identifying true obesity, BMI has a 0.743 sensitivity, a specificity of 0.648, a positive predictive value (PPV) of 0.738 and a negative predictive value (NPV) of 0.654 (Table 2).

**Table 2:** Classification of obesity by DEXA %BF versus classification of obesity by BMI

	Category by DEXA %BF		
Category by BMI	Nonobese	Obese	Total
Nonobese	387	205	592
Obese	210	592	802
Total	597	797	1394

Columns and rows refer to categories of obesity. The numbers represent the counts of individuals assigned to nonobese and obese groups. Sensitivity=0.743, specificity=0.648, positive predictive value (PPV)=0.738, negative predictive value (NPV)=0.654.

DEXA %BF, dual-energy X-ray absorptiometry percent body fat.

**Figure 2:**
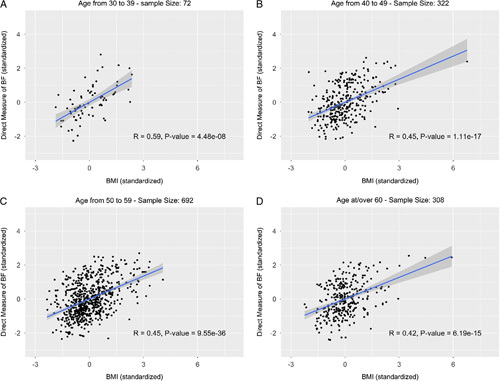
Direct measure of body fat (DEXA %BF) versus BMI by age group (standardized). (A) Age from 30 to 39 – sample size: 72; (B) age from 40 to 49 – sample size: 322; (C) age from 50 to 59 – sample size: 692; (D) age at/over 60 – sample size: 308.

We showed that as individuals age, BMI becomes a less reliable measurement. Figure 2 demonstrates that younger age groups align with a higher correlation between BMI and %BF. The Pearson correlation coefficient was clearly highest for those under 40 (*r*=0.59). This value decreased to 0.45 in the age group 40–49 and 50–59. Over 60, it decreased slightly to 0.42. This relationship was consistent among both males and females (Supplementary Figures 2, Supplemental Digital Content 4, http://links.lww.com/MS9/A45 and 3, Supplemental Digital Content 5, http://links.lww.com/MS9/A46).

We also examined the impact of gender on the accuracy of BMI. The correlation between BMI and DEXA %BF was 0.66 in female samples, which is greater than the 0.55 in male samples, while both correlations were statistically significant with a *P* value less than 0.001 (Fig. 1B, C). When using DEXA BF% in place of BMI, we found that 24% of all female patients were reclassified to a different tier, and 32% of all male patients were reclassified. There was a significantly higher proportion of male patients (16.9%) versus 10.1% of females who were overestimated by BMI as obese (*P*=0.003). The proportion of underestimated obesity by BMI (13.5% in females vs. 15.1% in males) was not statistically different (Table 3). The specificity of BMI is lower for males (0.62) than for females (0.76). We tested whether gender interacts with BMI in predicting DEXA %BF, and no significant interactions were identified (Supplementary Table 2, Supplemental Digital Content 6, http://links.lww.com/MS9/A47).

**Table 3:** Obesity classification by DEXA %BF and BMI by gender.

	N(D)N(B)^a^	N(D)O(B)	O(D)N(B)	O(D)O(B)	Total
Female	116 (30.77)	38 (10.08^b^)	51 (13.53)	172 (45.62)	377 (100)
Male	271 (26.65)	172 (16.91^b^)	154 (15.14)	420 (41.30)	1017 (100)

^a^ N(D)N(B): nonobese by DEXA %BF and BMI; N(D)O(B): nonobese by DEXA %BF and obese by BMI; O(D)N(B): obese by DEXA %BF and nonobese by BMI; O(D)O(B): obese by DEXA %BF and BMI.

The numbers represent the counts of individuals, while the numbers in the parentheses are the row percentages.

^b^ Two-proportions *z* test gave *P*=0.002 when comparing the two proportions.

DEXA %BF, dual-energy X-ray absorptiometry percent body fat.

**Figure 3:**
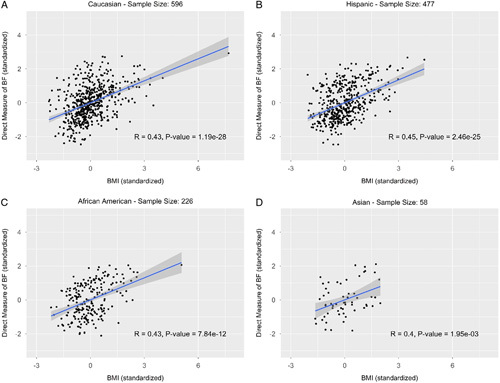
Direct measure of body fat (DEXA %BF) versus BMI by ethnicity (standardized). (A) Caucasian – sample size: 596; (B) Hispanic – sample size: 477; (C) African American – sample size: 226; (D) Asian – sample size: 58.

In the analysis of ethnic groups, the total sample size is less than the full sample size of 1394 because there were certain ethnic groups that had either a single or minimal representation, which would not allow for statistical analysis. Among 1394 samples, 1357 were from the four major ethnic groups – Caucasians, Hispanics, African Americans, and Asians (Fig. 3). The correlation coefficient was the highest in the Hispanic population with a value of 0.45 and the lowest in the Asian population with value 0.40. The correlation coefficient was 0.43 for both the Caucasian population and the African American population. All correlations were moderate but statistically significant with a *P* value less than 0.001 (Fig. 3). In the African American population, 25.2% of samples were overestimated, and 6.2% were underestimated by BMI, which were significantly different from the 13.6% overestimated and 17.5% underestimated in the Caucasian population with *P* values less than 0.001 (Table 4A). When the BMI cutoff for obesity was adjusted to 32 for the African American population, there were no longer statistically significant differences between African American samples as compared to other populations (Table 4B). AUC (area under the receiver operating characteristic curve) was in the range of 0.74–0.83 in all the groups we examined (Supplementary Figure 1, Supplemental Digital Content 2, http://links.lww.com/MS9/A43).

**Table 4:** Obesity classification by DEXA %BF (A) and BMI (B) by race.

	N(D)N(B)^a^	N(D)O(B)	O(D)N(B)	O(D)O(B)	Total
(A)					
Asian	13 (22.41)	9 (15.52)	7 (12.07)	29 (50)	58 (100)
African American	61 (26.99)	57 (25.22^b^)	14 (6.20^b^)	94 (41.59)	226 (100)
Hispanic	112 (23.48)	58 (12.16)	76 (15.93)	231 (48.43)	477 (100)
Caucasian	182 (30.54)	81 (13.59)	104 (17.45)	229 (38.42)	596 (100)
(B)					
Asian	13 (22.41)	9 (15.52)	7 (12.07)	29 (50)	58 (100)
African American	78 (32.30)	40 (19.91^c^)	29 (10.62^d^)	79 (37.17)	226 (100)
Hispanic	112 (23.48)	58 (12.16)	76 (15.93)	231 (48.43)	477 (100)
Caucasian	182 (30.54)	81 (13.59)	104 (17.45)	229 (38.42)	596 (100)

^a^ N(D)N(B), nonobese by DEXA %BF and BMI; N(D)O(B): nonobese by DEXA %BF and obese by BMI; O(D)N(B): obese by DEXA %BF and nonobese by BMI; O(D)O(B): obese by DEXA %BF and BMI.

The numbers outside the parentheses represent the counts of individuals, while the numbers in the parentheses are the row percentages in %.

(a) BMI obesity cutoff is 27.5 for the Asian population and 30 for the other populations; (b) BMI obesity is 27.5 for the Asian population, 32 for the African American population, and 30 for Hispanic and Caucasian populations.

^b^ Two-proportions *z* test gave *P*<0.001 compared to the proportions in the Caucasian population.

^c^ Two-proportions *z* test gave *P*=0.17 compared to the proportions in the Caucasian population.

^d^ Two-proportions *z* test gave *P*=0.13 compared to the proportions in the Caucasian population.

DEXA %BF, dual-energy X-ray absorptiometry percent body fat.

## Discussion

We noted that a high percentage of patients were obese, although this was not identified by BMI. This is consistent with previous studies showing that BMI can frequently misclassify patients. It is possible as we have become more sedentary, the same BMI measurement may be associated with a higher percentage of body fat now as compared to many years ago, due to a relative decrease in muscle mass.

This study showed that the correlation between an estimated BMI versus DEXA %BF is poor. BMI has long been used as an estimate for obesity, and its operating characteristics can justify its usage to a certain extent across various populations. However, individuals are often misclassified, which highlights the limitations of BMI as opposed to DEXA %BF.

The increase in the false categorization of older individuals as nonobese can be attributed to sarcopenic obesity^22^. As part of normal aging, there is a tendency for individuals to lose body muscle mass and have a higher percent body fat^23^. Since the BMI table does not adjust for age, cases of obesity will be systematically misclassified as nonobese or slightly overweight. The DEXA %BF will be particularly valuable in older patients.

In using *R* ^2^, we highlight the variance between DEXA %BF and BMI. While BMI may properly reflect national obesity rates on average, it poorly reflects the variation present from person to person. This deficiency of BMI becomes more problematic with increasing age.

There are many studies that suggest the need to change the BMI cutoff for the Asian population to 27.5 from 30^24^. Changing the cutoff did not significantly improve BMI’s accuracy of obesity evaluation within our Asian population bracket.

Overall, the measurements and correlation seen in the African American population are significantly statistically different from the other ethnic groups, confirming the observation that a higher BMI cutoff to classify obesity is appropriate for the African American population^25^. Perhaps this would suggest that just as there is clinical data supporting changing the BMI cutoff for Asians, the same could be said for the black population as well. This may suggest that a BMI of 30 is low for black populations, perhaps due to this group having a higher muscle mass.

We previously reported a study on DEXA %BF and BMI in a population of National Football League (NFL) football players, which is consistent with the current analysis. In the prior study, the average age was 42, with 80% of the population composed of African Americans. In this paper, the statistics led us to believe that the only way to be certain that an NFL player was obese was to have a BMI over 40. It would be clinically beneficial to find a better cutoff number for the black population. We found in our earlier paper looking at retired football players, predominantly black, BMI significantly overestimated the rate of obesity. A study of college athletes that compared DEXA %BF to BMI also found BMI overestimated obesity. Because of the confounding of race with muscle mass, we cannot definitively determine how much variation is attributed to race versus muscle mass.

## Limitations

This study was done at a single center on worker’s compensation patients which may limit the generalizability of these results to other patient populations. Not all patients agreed to scanning, so there is a possibility of some selection bias, though we identified this occurred in only five patients. We did not prospectively collect details as to the amount of labor they were doing on their jobs, which might have been of interest to see if classification varied based on occupational status.

While there is a clear trend that BMI tends to be a better predictor in the female population than the relevant male population, our study does have a limited female population. The sample size for the female population was significantly smaller than the male population. Because of the slight radiation exposure that accompanies measuring DEXA %BF, women were not scanned as often. This phenomenon contributes to the smaller population size in females overall, especially in the younger age groups.

## Conclusions

We found that BMI misclassified obesity in a significant percentage of the population. We did show that DEXA %BF in routine clinical practice provides more precise information to guide clinical decision-making. Because of the magnitude of this difference, the performance of the DEXA scan should be considered for broader clinical use. It might be particularly valuable in men, Blacks, or Asians, who are found to have a higher rate of discordance between BMI and DEXA %BF.

## Ethical approval

Not applicable.

## Patient consent

Not applicable – retrospective chart review. Consent is given for examination and dissemination of results obtained.

## Source of funding

HymanHealth Inc. and the National Center for Advancing Translational Sciences, CTSI grant UL1TR001881, and the National Institute of Diabetes and Digestive and Kidney Disease Diabetes Research Center (DRC) grant DK063491 to the Southern California Diabetes Endocrinology Research Center.

## Author contribution

All contributors provided equal input. Ms Peled did the majority of data extraction from the charts.

## Conflicts of interest disclosure

There are no conflicts of interest.

## Guarantor

Mark H. Hyman, MD.

## Clinical Statement

Obesity is a major determinant of health. Correctly identifying patients with obesity is important for recommending strategies to reduce the disease burden that accompanies the obese state. We assessed the accuracy of defining obesity in a worker compensation population. More definitive measures of Obesity are needed in reporting and interpreting Obesity study results.

## Provenance and peer review

Not commissioned, externally peer-reviewed.

## Supplementary Material
