# Supplementary figures and images for: Differences in BMI obesity measures in a workers compensation population: a cross-sectional study
Source: Ann Med Surg (Lond). 2023 Apr 1;85(5):1607–13. doi: 10.1097/MS9.0000000000000428 (PMC10205388; doi:10.1097/MS9.0000000000000428)

Supplementary Figure 1. ROC of Direct Measure of Body Fat (DEXA%BF) versus BMI
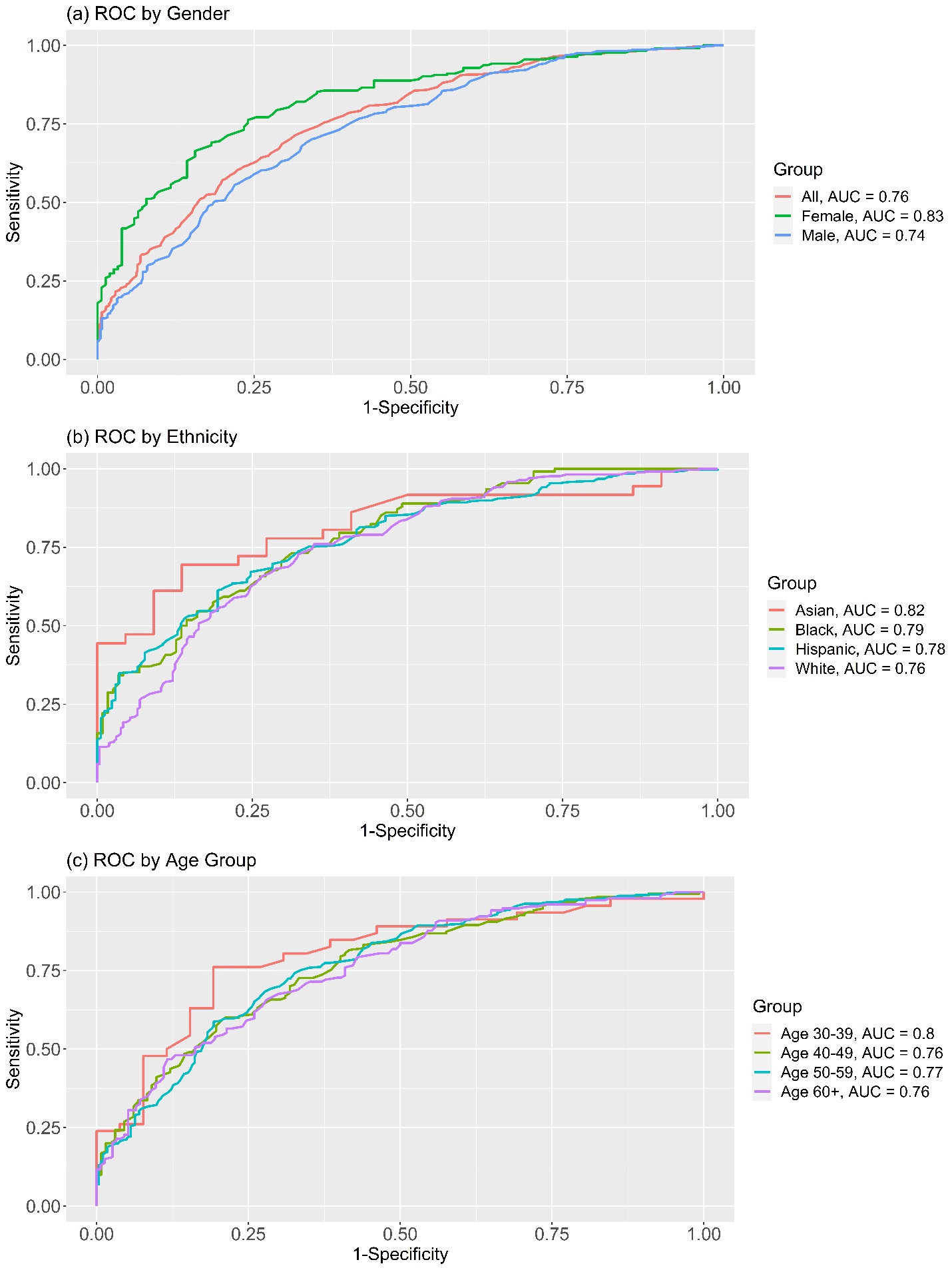

Supplement: Supplementary file 2 [file ms9-85-1607-s002.docx]
