# Supplementary material for: Differences in BMI obesity measures in a workers compensation population: a cross-sectional study
Source: Ann Med Surg (Lond). 2023 Apr 1;85(5):1607–13. doi: 10.1097/MS9.0000000000000428 (PMC10205388; doi:10.1097/MS9.0000000000000428)
Supplement: Supplementary file 3 [file ms9-85-1607-s003.docx]

| Supplementary Table 1. Sensitivity Analysis | | |
| --- | --- | --- |
| Sample | Correlation | p_value |
| all | 0.44 | 3.37E-67 |
| sub1 | 0.43 | 1.52E-64 |
| sub2 | 0.42 | 1.17E-58 |
| sub1: removed 2 outliers with BMI that are ≥ 5 sd away from the mean | | |
| sub2: removed 12 outliers with BMI that are ≥ 3 sd away from the mean | | |
