# Supplementary material for: Differences in BMI obesity measures in a workers compensation population: a cross-sectional study
Source: Ann Med Surg (Lond). 2023 Apr 1;85(5):1607–13. doi: 10.1097/MS9.0000000000000428 (PMC10205388; doi:10.1097/MS9.0000000000000428)

Supplementary Figure 3. Direct Measure of Body Fat (DEXA%BF) versus BMI by Age Group in Females (standardized)


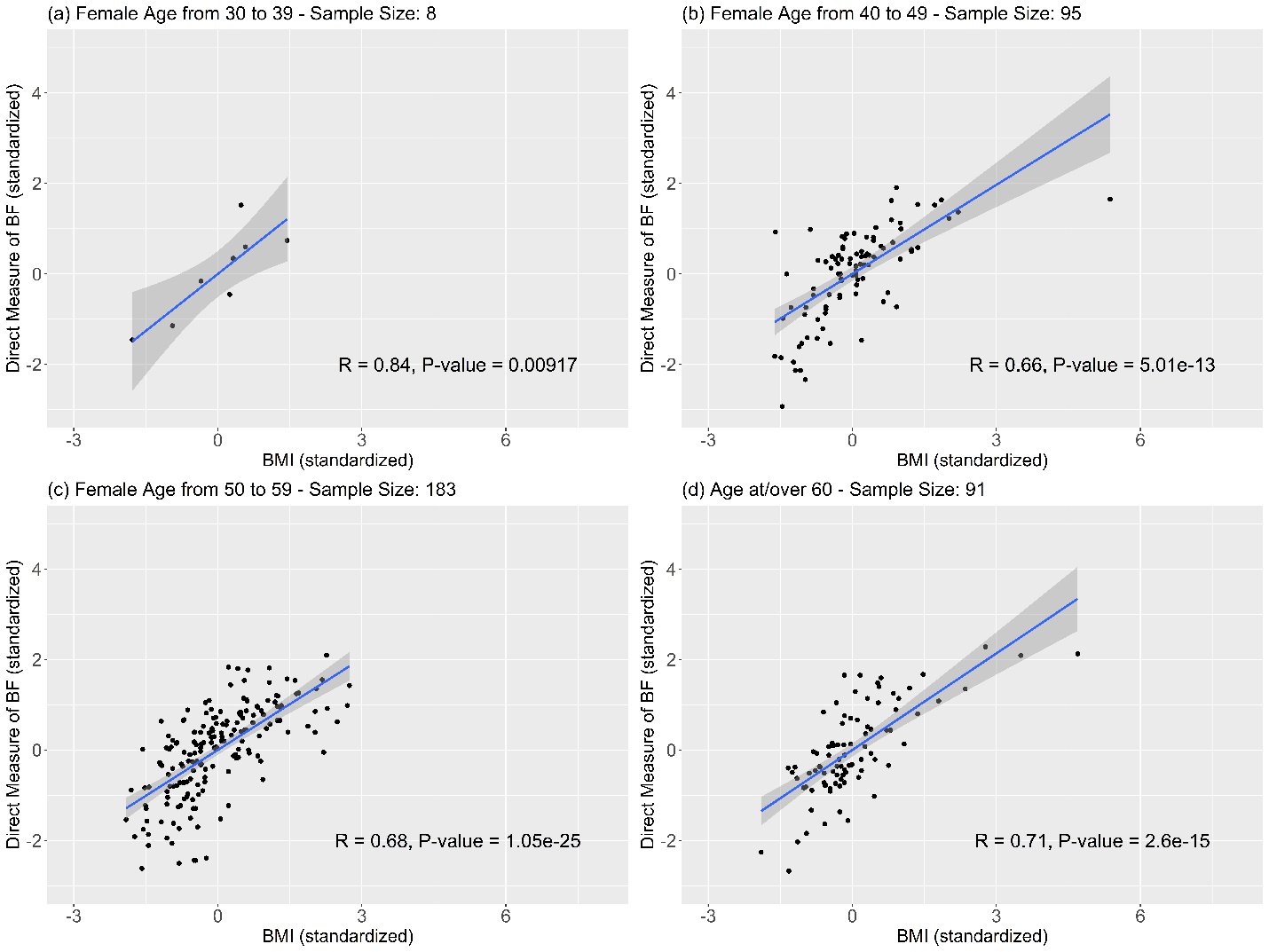

Supplement: Supplementary file 5 [file ms9-85-1607-s005.docx]
