# Supplementary material for: Differences in BMI obesity measures in a workers compensation population: a cross-sectional study
Source: Ann Med Surg (Lond). 2023 Apr 1;85(5):1607–13. doi: 10.1097/MS9.0000000000000428 (PMC10205388; doi:10.1097/MS9.0000000000000428)
Supplement: Supplementary file 6 [file ms9-85-1607-s006.docx]

| Supplementary Table 2. Interaction analysis results | | | |
| --- | --- | --- | --- |
| Sample | P_value | | |
|  | bmi_z | male | bmi_z*male |
| All | 9.43E-54 | 5.89E-246 | 1.61E-01 |
| Asian | 3.53E-04 | 3.58E-14 | 3.66E-01 |
| Hispanic | 2.20E-23 | 1.22E-97 | 5.61E-01 |
| Black | 1.66E-16 | 1.32E-61 | 5.40E-01 |
| White | 1.44E-12 | 2.26E-62 | 4.39E-02 |
| age30-39 | 4.96E-03 | 2.49E-10 | 7.14E-01 |
| age40_49 | 2.01E-16 | 2.43E-51 | 9.22E-01 |
| age50_59 | 3.48E-29 | 1.55E-131 | 1.16E-01 |
| age60 + | 3.44E-12 | 3.24E-60 | 7.05E-01 |
